# Evaluation of a complex intervention to improve activities of daily living of disabled cancer patients: protocol for a randomised controlled study and feasibility of recruitment and intervention

**DOI:** 10.1186/1472-6963-14-194

**Published:** 2014-04-29

**Authors:** Line Lindahl-Jacobsen, Dorte Gilså Hansen, Karen la Cour, Jens Søndergaard

**Affiliations:** 1 Research Unit of General Practice, Institute of Public Health, University of Southern Denmark, JB Winsløws Vej 9A, Odense C 5000, Denmark; 2 Department of Rheumatology and Rehabilitation, Næstved Hospital, Region Zealand, Ringstedgade 61, Næstved 4700, Denmark; 3 Research Initiative of Activity Studies - Health, Man and Society, Institute of Public Health, University of Southern Denmark, JB Winsløws Vej 9B, Odense C 5000, Denmark

**Keywords:** Cancer rehabilitation, Occupational therapy, Activities of daily living, Intervention studies, Feasibility studies

## Abstract

**Background:**

Many cancer patients have problems performing activities of daily living (ADL). A randomised controlled trial was designed to examine the effects of an ADL intervention in addition to standard treatment and care in a hospital setting. The objective of this article was to present the study and to analyse the feasibility of the recruitment process and the intervention.

**Methods:**

Adult disabled cancer patients at Næstved Hospital in Denmark were enrolled between 1 March 2010 and 30 June 2011 and randomised into an ADL intervention or to a control group. The intervention was performed by occupational therapists. The feasibility of the recruitment was analysed with regard to success in achieving the estimated number of participants and identification of barriers, and feasibility of the intervention was based on calculations of patient attendance and patient acceptability. The primary outcome of the randomised controlled trial was patients’ health-related quality of life 2 and 8 weeks after baseline.

**Results:**

A total of 118 disabled cancer patients were enrolled in the study over a time span of 16 months. Very few meetings between occupational therapist and patient were cancelled. Time spent on the intervention varied considerably, but for the majority of patients, time consumption was between 1–3 hours.

**Conclusions:**

Despite difficulties with recruitment, participation was considered feasible and the intervention was accepted among patients. Missing data in the follow-up period were mostly due to death among participants. Very few participants declined to complete questionnaires during follow-up.

## Background

Activities of daily living (ADL) is a concept often used in relation to the things we normally do in our lives and constitute everyday tasks carried out in order to sustain a controllable and meaningful life [1]. ADL can be divided into self-care (personal care, functional mobility, community management), productivity (work tasks, household management, play/school) and leisure activities (sports, hobbies and participation in social activities) [2]. This paper reports experiences from an intervention study with focus on ADL. Cancer patients were selected on the basis of the Karnofsky Performance Status Scale and comprised disabled patients in need of help in their daily lives.

Many cancer patients experience physical, psychological and social problems during and after cancer treatment, including difficulties with ADL [3]. Patients with persistent cancer disease tend to have more problems with ADL compared to cancer-free controls [4]. Previous research has found that cancer patients have a feeling of relative well-being when they are able to perform activities in daily life [5], but it is unknown whether ADL performance is associated with health-related quality of life among disabled cancer patients. It is assumed that interventions targeting ADL performance may be effective in handling some of the cancer patients’ problems, but evidence is sparse. In Denmark, as in many other countries, such interventions are often carried out by occupational therapists. Previous studies on occupational therapy and cancer have primarily been observational and suggest that people living with life-threatening illness use meaningful occupations to regain a sense of control and normality in their lives [5]. To our knowledge the only published randomised controlled trial evaluating the topic investigated an intervention aimed to reduce limitations in daily activities in rural breast cancer survivors. They found that a telephone-based problem-solving occupational therapy intervention programme was feasible and had positive effects on function, quality of life and emotional state. However, the study involved only breast cancer patients and a very small sample [6]. Previous studies aiming to evaluate occupational therapy to cancer patients have either to a limited extent focused on ADL or used a descriptive approach [7-10], comprised very few patients [9,11], been a pilot study [6,12] or had focus on creative and social activities [13]. There is evidence that occupational therapy is an effective intervention to improve ADL in stroke patients [14,15], elderly people and in patients with rheumatoid arthritis [14]. Occupational therapy targeting ADL can increase performance score and even decrease deaths and deterioration of ADL in patients with stroke [15], improve functional ability in patients with stroke, rheumatoid arthritis and elderly people, increase participation among patients with stroke and elderly people [14] and improve well-being in elderly people [14,16,17].

Hence, there is a lack of rigorously conducted randomised controlled trials (RCT) aiming to improve ADL of cancer patients, irrespective of cancer type.

### Objectives

A randomised controlled trial with focus on the effects of an ADL intervention among disabled cancer patients was designed to test the hypothesis that a targeted ADL intervention could enhance participation in everyday activities and improve quality of life of patients with cancer.

This paper aims to 1) describe the intervention and the recruitment strategy, 2) analyse the feasibility of patient recruitment, and 3) evaluate the intervention, i.e. the implementation process and setting.

## Methods

### Design

**Figure 1:**
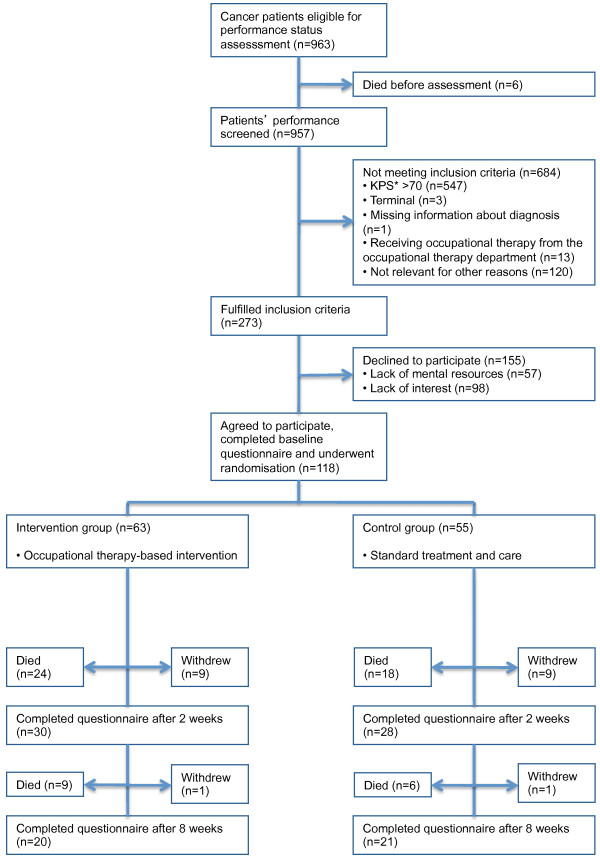
**Recruitment of patients to the RCT on occupational therapy intervention.** *KPS: Karnofsky Performance Status.

The study was designed and performed in accordance with the CONSORT statement [18] and within the framework for the development and evaluation of complex interventions [19]. In order to provide an overview of the recruitment process, the intervention and follow-up, Figure 1 shows an outline of the process [19].

From 1 March 2010 to 30 June 2011 we enrolled inpatients and outpatients with cancer in a non-blinded RCT on the added value of ADL intervention. The overall goal of the ADL intervention was to increase patients’ quality of life and ability to live an independent and meaningful life by playing an active part in relation to ADL.

This study was registered at ClinicalTrials.gov with the registration ID number: NCT01432197.

### Setting

The study was conducted at Næstved Hospital, Denmark, in 4 different departments - 3 inpatient departments: a gastrointestinal surgical department, a lung disease department, an oncology department, and one oncology outpatient clinic. Næstved Hospital is a public general hospital in Region Zealand (820 000 inhabitants) and constitutes one of two hospitals in the region with clinical oncological specialty. The majority of cancer patients treated at Næstved Hospital have either breast, lung, colorectal, pancreatic, bladder or blood cancer. During the study period two occupational therapists from the occupational therapy department were employed full-time to conduct the recruitment and intervention. The occupational therapy department comprised 18 occupational therapists working in the fields of neurology, orthopedic surgery, paediatrics, geriatrics, medical diseases and oncology.

The Danish healthcare system is publicly funded and ensures free access to in- and outpatient care for all citizens.

### Recruitment of study participants

Cancer patients (≥18 years) were eligible to participate if they 1) had a pathologically confirmed cancer diagnosis, regardless of cancer type, 2) had disabilities comparable with a performance status score of 10 to 70 on the Karnofsky Performance Status Scale (KPS), 3) were not referred to occupational therapy prior to study start, and 4) were able to read and understand Danish and fill in questionnaires. Participants were randomly assigned to one of the two groups. The control group was given standard treatment and care, and the intervention group was given the ADL intervention as a supplement to standard treatment and care. Patients in the intervention group met with an occupational therapist various times in an inpatient or outpatient setting, depending on their individual needs (Figure 1).

In cooperation with nurses at the respective departments, the two occupational therapists collected patient lists including information about cancer patients present at the four departments on every weekday and scored each patient’s performance status with the KPS. The occupational therapists also performed the intervention and took part in the data collection.

### The intervention

**Figure 2:**
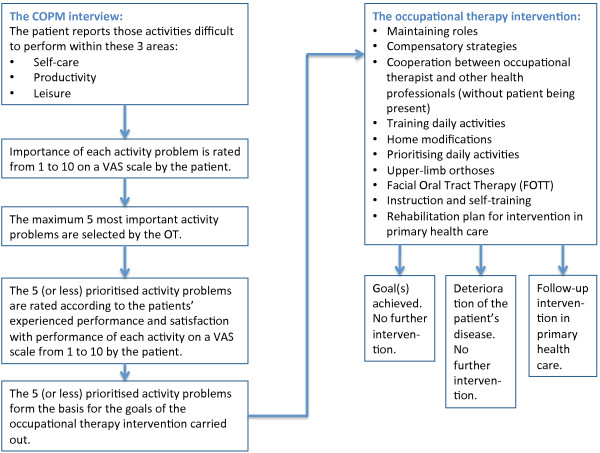
Decision diagram followed by the occupational therapists in conducting the intervention.

The intervention was based on a patient-centred assessment of problems with activities of daily living, i.e. personal, work and leisure activities. The intervention took place at the hospital or in the patients’ homes and included different types of ADL interventions (Table 1). In the first session the patients were assessed systematically for problems and rehabilitation needs during an interview based on the Canadian Occupational Performance Measure (COPM) [2]. The interview focused on the patient’s ability to carry out personal, work and leisure activities, and a prioritised list of rehabilitation needs was made in agreement between patient and occupational therapist, including a targeted tailored plan of support (Figure 2). To meet the needs of the individual patient, the intervention was flexible in number of sessions. In joint negotiation the occupational therapists and the patients assessed the number of sessions needed, based on when the patients’ goals were met.

**Table 1:** Different components of the ADL assessment and intervention tested in the RCT study: activity, content and examples

**Activity**	**Content**	**Examples**
**Maintaining roles**	• Patient informs about family pattern	• Roles in the patient’s family
	• Occupational therapist advises patient in maintaining roles despite illness	• Using assistive devices
**Compensatory strategies**	• Ergonomics	• Advice on work techniques
	• Information about energy-saving methods	
	• Assistive devices	• Delivery
		• Instruction	
		• Supervision on use	
**Cooperation between occupational therapist and other health professionals (without patient being present)**	• Practical matters in the departments	• Conferences	
	• Arranging help in patient’s home after discharge	• Telephone calls	
	• Referral to local specialists for support and treatment, i.e. GP, physiotherapist, occupational therapist	• Planning the intervention	
**Training daily activities**	• Personal activities	• Dressing	
	(occupational therapist observes and guides the patient)	• Bathing	
	• Occupational and household activities	• Work	
	(occupational therapist observes and guides the patient)	• Housework	
	• Leisure activities	• Swimming	
	(occupational therapist observes and guides the patient)	• Watching TV	
**Home modifications**	• Adapting the environment to meet the patient’s needs.	• Refurnishing	
		• Installation of ramps	
		• Installation of raised toilet seat	
		• Rearranging the room functionality	
**Prioritising daily activities**	• Occupational therapist assists the patient in prioritising daily activities	• Receiving assistance with some activities	
		• Sustaining self-reliance in the most important activities	
**Upper-limb orthoses**	• Adjustment	• Hand orthoses	
	• Supply	• Lymphedema gloves	
**Facial Oral Tract Therapy (FOTT)**	• Specific analysis of difficulties handling eating procedures	• Cleaning teeth	
	• Posture, movement, sensation	• Eating	
	• Exercises	• Drinking	
**Instruction and self-training**	• Instruction in transfer	• Accessibility	
		• Mobility	
	• Self-training programmes	• ADL training	
		• Physical training	
		• FOTT exercises	
**Rehabilitation plan for intervention in primary health care**	• Rehabilitation plan is made in cooperation between patient and occupational therapist and sent to therapists in primary health care	• Functional level at time of discharge	
		• Rehabilitation goals	

Different elements of the intervention included training of ADL, home modifications, supply of and supervision in the use of adaptive equipment for use during hospitalisation and at home, assisting the patient in prioritising between ADL and instruction in self-training programmes and transfers (accessibility and mobility in the home environment) (Table 1). Special emphasis was put on changes in patients’ needs, and priorities and goals/plans were continuously revised. In agreement with the patients, relatives were involved when relevant.

The need for support and treatment from other health professionals in the local community as well as at the hospital, for example occupational therapist, physiotherapist, oncologist, nurse or general practitioner, was continuously evaluated and facilitated.

The two occupational therapists conducting the intervention had a broad clinical experience within cancer rehabilitation, in supportive as well as palliative care. Prior to study start a protocol describing the intervention in detail (activities shown in Table 1) was made. The intervention was developed based on guidelines from HOPE; The College of Occupational Therapists’ Section for HIV/AIDS, Oncology, Palliative Care and Education, generated from experiences from more than 40 British specialist practitioners combined with existing literature on occupational therapy for cancer patients. The guidelines provide information on what occupational therapy can do for specific symptoms associated with cancer. They cover patients with all types of cancer in all parts of the cancer pathway from diagnosis through treatment, long-term monitoring, follow-up, relapse, cure, palliative care and terminal care in different settings, i.e. hospital, hospice, nursing home or patient’s own home [20]. The occupational therapists participated in a programme including specific instructions with regard to conducting the study intervention. All procedures from recruitment and intervention to follow-up were strictly monitored [19] by completion of forms, and the occupational therapists closely followed the protocol of clinical instructions for performing the intervention programme during the study period.

### Control group

Patients randomly assigned to standard care were not scheduled to meet any occupational therapist, unless a nurse or oncologist in one of the departments sent a referral to the occupational therapy department. Controls thus referred to occupational therapy at the hospital during the study period did not cross over to the intervention group. Standard care comprised routine medical treatment, nursing, psychological treatment and physiotherapy.

In Denmark occupational therapy is not yet an integrated part of cancer patients’ treatment, rehabilitation and palliation.

### Outcomes and data collection

Patient data and evaluation were based on patient questionnaires at baseline and 2 and 8 weeks after. A long follow-up period was preferred, but considering a potentially large mortality due to the condition of the patients it was decided to make it no longer than 8 weeks*.* To evaluate the effect of the intervention the primary outcome of the RCT was patient-perceived health-related quality of life measured by the Global Health Status items at the European Organisation for Research and Treatment of Cancer Quality of Life Questionnaire C30 (EORTC QLQ-C30) [21] at 2 and 8 weeks after randomisation. Secondary outcome was the patient’s ability to perform ADL measured by the ADL Taxonomy Questionnaire [22] at 2 and 8 weeks after baseline.

The outcomes of the feasibility of recruitment were number of participants relative to sample size estimations and identification of the barriers. The outcomes of the feasibility of the intervention were 1) success in implementing the intervention, 2) patient attendance, and 3) acceptance of intervention.

Baseline questionnaires including information on demographics, ADL and quality of life were completed at the hospital, and follow-up questionnaires including ADL and quality of life were sent by mail and returned in a prepaid envelope. Non-respondents received a reminder (questionnaire and prepaid envelope) after four weeks.

Register data on diagnosis, number of sick days and contact to other healthcare professionals such as general practitioner and physiotherapist was obtained.

### Sample size

The sample size was estimated based on the primary outcome measure. According to the EORTC Tables of Reference Values in a mixed cancer population the Global Health Status is normally distributed with a mean of 61.3 and an SD of 24.2. A change of 8 units was assumed to be clinically relevant. With a power of 80% the sample size was calculated to be 144 patients in each group [23].

### Randomisation

The occupational therapists contacted a secretary not otherwise involved in the study, who conducted the randomisation using the SiMin software. To ensure an equal distribution of patients with low and intermediate performance in the two groups, participants were stratified based on their KPS score (low: 10–40 and intermediate: 50–70) using the minimisation procedure [24-26]. Minimisation is a procedure ensuring that treatment groups are similar with respect to a series of predefined prognostic factors. As patients are recruited to the trial they are allocated to the treatment groups that will minimise the differences in the distribution of those factors between the groups [24,25], and even in small trials it will provide groups that are very similar on several prognostic factors [25]. Assignment was evident to the patients and the occupational therapists, but was blinded to the researchers.

### Piloting of recruitment process, questionnaires and intervention

To test the recruitment process, the logistics and the oral and written communication [19], we spent one month recruiting 20 patients to a pilot study, including interviews about problems of ADL and rehabilitation goals based on the COPM. The patients were either cancer patients referred to the occupational therapy department or cancer patients from 2 of the 4 departments. As a result minor adjustments to the written information were made and we decided to cooperate with 2 additional departments in order to improve the recruitment.

The questionnaires were first sent to a group of researchers without direct relation to the study to receive comments about content, layout, scope and applicability. Secondly, the occupational therapists in the occupational therapy department were asked to fill in the questionnaires and comment on layout. Thirdly, the pilot group of patients filled in the questionnaires. Minor corrections to the *ad-hoc* part of the questionnaire were made.

From the pilot phase we further developed the logistics in identifying the patients in the departments and learnt that the communication was more time-consuming than expected.

### Analysis

To analyse the flow of patients during recruitment we calculated the number of cancer patients assessed by the occupational therapists, the relative number of patients who were eligible and the relative number of study participants of patients eligible for the study. To test for inclusion bias we compared participants with non-participants with regard to cancer type, age and sex.

We analysed the time spent on the recruitment process and estimated the expected duration of inclusion procedure to reach the number of participants calculated from the power estimation.

**Figure 3:**
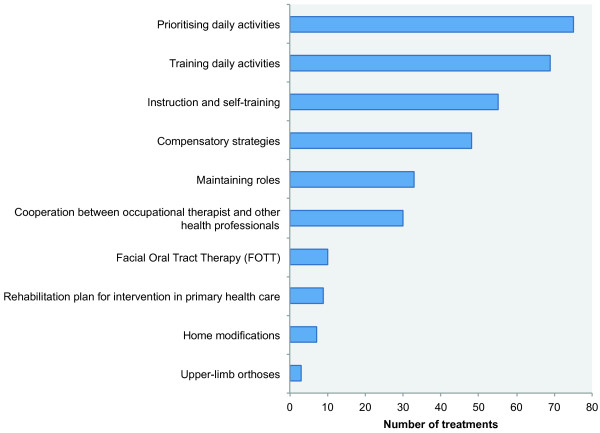
Distribution of activities following the COPM interview within the occupational therapy intervention in the RCT, N = 339 to 63 patients.

**Figure 4:**
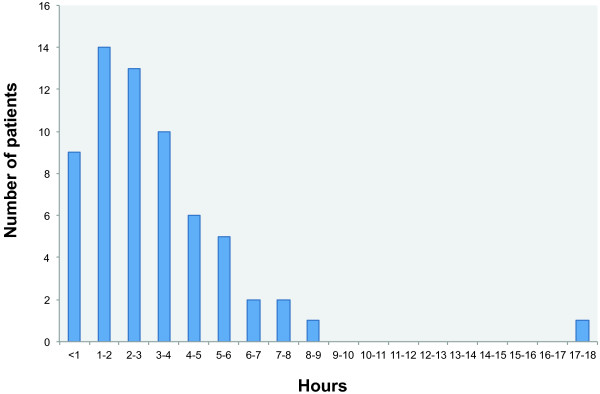
Total time consumption of occupational therapy intervention by patients.

To assess the feasibility of the intervention, for each patient in the intervention group we registered what kind of interventions were given (Figure 3), the time spent on the intervention (hours) (Figure 4), where it took place (ward/outpatient clinic/patient’s home), and whether the intervention had led to further actions, i.e. interventions in primary health care.

### Ethics

The Danish Data Protection Agency and the Regional Ethics Committee on Biomedical Research (ID: 1-01-83-0002-07) approved the study. All participants received oral and written information about the study and all patients provided written informed consent.

## Results

### The recruitment process

Flowchart of recruitment and follow-up is presented in Figure 1. During 16 months a total of 963 cancer patients were identified, 6 patients died and 957 were assessed for eligibility. Of these, 684 had a performance status above the level or were not eligible for other reasons, i.e. being terminally ill, receiving occupational therapy in the occupational therapy department at time of inclusion, being unable to participate due to cognitive problems or poor language skills, or based on nurses’ opinion too ill.

Of the 273 who fulfilled the inclusion criteria 155 (56.8%) did not want to participate. In total 118 (43.2%) patients were included and randomised, 63 to the intervention group and 55 to the control group (Figure 1). This minor imbalance between number of participants in the two groups was most probably due to chance. According to the sample size calculation the number of patients should be 144 in each group, 288 in total. Despite dedicated efforts to recruit a sufficient number of patients for the study, the recruitment and inclusion were more time-consuming and slower than expected. Two occupational therapists spent 16 months assessing and recruiting the 118 patients for the study and conducting the intervention. Due to financial and local conditions the inclusion was terminated following inclusion of a number of patients considerably below sample size estimations. Estimations following first year of inclusion showed that an average of 7.25 (range: 1–17) patients were recruited each month, leading to a study period of 40 months to reach the estimated 288. With the 118 participants actually obtained in this study and with the assumed statistical power and variance from the original sample size calculation we are able to detect a difference in mean Global Health Status between the intervention group and control group of 12.5.

The most frequent cancer diagnoses of participants were lung cancer (31.4%), breast cancer (17.8%), colon cancer (15.3%), pancreatic cancer (6.8%) and rectal cancer (5.9%). These results were almost similar to those of the non-participants. Non-participants were generally older than participants, and while the distribution of men and women was almost similar in non-participants, two-thirds of the participants were women (Table 2).

**Table 2:** Participants and non-participants

**Variable**	**Participants, n = 118 (100%)**	**Non-participants, n = 155 (100%)**
**Sex**		
Women	77 (65.3%)	77 (49.7%)
Men	41 (34.8%)	78 (50.3%)
**Age**		
30-50 years	5 (4.2%)	9 (5.8%)
51-70 years	67 (56.8%)	51 (32.9%)
71-90 years	45 (38.1%)	90 (58.1%)
>90 years	1 (0.9%)	5 (3.2%)
**Cancer types**		
Lung cancer	37 (31.4%)	50 (32.3%)
Breast cancer	21 (17.8%)	22 (14.2%)
Colon cancer	18 (15.3%)	29 (18.7%)
Pancreatic cancer	8 (6.8%)	7 (4.5%)
Rectal cancer	7 (5.9%)	17 (11.0%)
Myeloma	7 (5.9%)	2 (1.3%)
Non-Hodgkin’s Lymphoma	6 (5.1%)	8 (5.2%)
Ovarian cancer	2 (1.7%)	2 (1.3%)
Melanoma	1 (0.8%)	2 (1.3%)
Leukemia	1 (0.8%)	7 (4.5%)
Endometrial cancer	1 (0.8%)	0 (0.0%)
Prostate cancer	0 (0.0%)	2 (1.3%)
Other	9 (7.6%)	7 (4.5%)

Of the 155 patients, who declined to participate in the study, 57 (36.8%) primarily reported that they lacked resources to participate, i.e. suffered from fatigue. A total of 98 (63.2%) reported lack of interest because they felt they had the help they needed in their daily lives (Figure 1).

Among the cancer patients enrolled in the study the majority rated their overall quality of life at baseline in the poor end of the EORTC QLQ-C30 (Table 3).

**Table 3:** Overall quality of life among study participants, global health status of the EORTC QLQ C-30

**Overall quality of life**	**N**	**%**
**1. Very poor**	12	10.2
**2.**	22	18.6
**3.**	29	24.6
**4.**	28	23.7
**5.**	17	14.4
**6.**	6	5.1
**7. Excellent**	2	1.7
**Unstated**	2	1.7
**Total**	118	100.0

### The intervention process

The patients in the intervention group (N = 63) were initially supposed to be interviewed based on the occupational performance measure (COPM) to agree on the individual intervention goals. This interview was carried out with 55 out of 63 (87%) patients in the intervention group. For the remaining 8 patients the subsequent intervention was based only on ADL observations, rather than rehabilitation goals identified through a COPM interview.

The largest proportion of the intervention took place at the hospital, except for home modifications, which of course took place in the patients’ homes. For the patients recruited from the outpatient clinic, the intervention typically took place at the occupational therapy department on days where they otherwise had appointments for ambulatory treatment. These patients either arranged their own transport to hospital or were transported by patient transport services. Only 10 out of 339 (2.9%) appointments for treatment by occupational therapist were cancelled by patients. The time spent on the entire intervention (including the COPM interview) for each patient varied from less than one hour to 18.5 hours, but for the majority of patients, time consumption was 1–3 hours (Figure 4). At the end of each session the type of intervention delivered was registered. Most treatments consisted of prioritisation and training of daily activities, while very few treatments consisted of supply and adjustment of upper-limb orthosis (Figure 3).

All patients enrolled completed the baseline questionnaire, 63 from the intervention group and 55 from the control group. No patients in the intervention group withdrew from the intervention programme. During follow-up, 30 from the intervention group and 28 from the control group completed questionnaire 2 (2 weeks after baseline) and 20 from the intervention group and 21 from the control group completed questionnaire 3 (8 weeks after baseline). The reason for not filling in the questionnaires in the follow-up period was primarily death (N = 56), since only 10 participants in each group withdrew due to other reasons (Figure 1).

There were not registered more referrals to occupational therapy from the four participating departments during this intervention period compared to earlier periods.

## Discussion

In this study based on 118 cancer patients we demonstrate that although we did not succeed in meeting the sample size calculation, it is feasible to recruit cancer patients for an intervention tailored the performance of everyday activities. The results from this study showed that despite knowledge on eligible patients and control of patient flow the recruitment process was more time consuming than expected. It took longer than expected to identify and contact potentially eligible patients in the respective departments, mostly because patients were preoccupied at the time, away from the ward due to CT scans, examinations etc., or because of delay due to uncertainty about the patients’ diagnoses. A significant proportion of eligible participants withdrew from the study when they were introduced to the written information on the study. The scope of information and formalities in the informed consent may have led patients to feel that participation in the study was too demanding.

The recruitment rate among eligible patients was 43.2%, and even though nearly 60% chose not to participate and argued that they might not need this kind of support, more than 40% still experienced a need and also decided to take part in the study. This is consistent with other intervention studies regarding cancer rehabilitation [27-29]. Among studies on cancer rehabilitation it is quite common to attain recruitment rates at about 20% [30-32]. Still, with a recruitment rate around 40% as in the present study we are aware of the fact that any results need to be extrapolated to a larger population and that this process needs to be performed carefully.

On average we recruited 7.2 patients per month. In the pilot study we recruited 20 patients over a period of one month. These patients were either recruited from the wards or referred to the occupational therapy department. The fact that we included patients already referred to the occupational therapy department probably made us overestimate the number of potential participants and assume a higher intake per month based on the monthly intake in the pilot study. There is a tendency towards recruitment rates of clinical studies on breast cancer patients being somewhat higher [33], compared with studies examining effects of new medicine to improve survival of cancer. Several other intervention studies were recruiting cancer patients at the respective departments at Næstved Hospital in the same time period. When patients are invited to participate in different projects, they may prioritise participating in studies aiming to improve their survival chances, rather than in studies focusing on participation in daily activities, or in the one they are presented with first. Improvements in both medical and surgical cancer treatment have resulted in more people surviving or living longer with their cancer disease, and it is therefore increasingly relevant to focus on the quality of life among those patients, who are going to live with a cancer disease for a long time. Health professionals face the challenge of being able to convey the utility of research that does not directly deal with survival.

Among the patients not wanting to participate in the study 63% reported lack of interest as the primary reason. Same explanation was found in another similar study, where lack of interest was present to the same degree [34]. It may de difficult to understand why so many patients did not want to participate in an intervention with focus on improving daily activities, but lack of knowledge on the concept ADL might be a reason, just like lack of resources due to anxiety and pain could be other potential reasons. Patients not wanting to participate did not differ significantly from participants in terms of diagnosis. The non-participants seemed to be somewhat older than those willing to participate, and women were more likely to participate than men (Table 2). The fact that the participants were younger than those declining to participate could indicate that the weakest patients chose not to enter the study. Despite this healthy worker effect, the majority of participants rated their overall quality of life in the poor end, indicating that they had potential to benefit from the intervention offered. Yet, another study found that those declining to participate were healthier than the participants [33].

The two occupational therapists clearly expressed the feasibility of following the protocol to provide the intervention. Results from monitoring the intervention demonstrate that the ADL intervention programme was easily tolerated and completed in the intervention group.

The two occupational therapists worked full-time on the project, which was crucial for completing identification of eligible patients, as well as for the clinical work in conducting the intervention. Implementation of the COPM interview succeeded in 87% of the participants in the intervention group, and therefore the subsequent intervention was generally based upon the patients’ personal needs and goals. Only few sessions between patient and occupational therapist were cancelled (3.5%), indicating the patients’ acceptance of the intervention as well as the success of the logistics. Because we could not blind the treatment, it cannot be ruled out that any possible effects of the intervention were the result of the supportive approach received by patients in the intervention group.

According to the sample size calculation the study population was estimated to be 288 patients, 144 in each group. The recruitment process stopped after 16 months due to financial matters and local conditions, and we succeeded in including a total of 118 cancer patients, substantially less than estimated by sample size calculations. However, the lower number of participants only leads to a 4.5 points reduction in detection level of the Global Health Status ranging from 0 to 100 [35].

Part of the recruitment strategy relied on nurses’ approval in terms of assessing the patients’ performance status. Steinhauser et al. [36] described the challenge of using healthcare providers as gatekeepers and suggested that physicians, nurses and other healthcare professionals may negatively affect recruitment as a result of inaccuracies in predicting time to death, protection of patients, inaccuracy in gauging the patients’ receptiveness to the research, or by allowing their personal opinions about the study’s benefit to affect referral. Experiences from this study showed how ‘ownership’ is essential when recruiting patients into a complex intervention, and the fewer intermediaries the better. Even though both leaders and managers were involved in facilitating this project, we learned that it is even more important to make efforts to continuously motivate those who take part in the recruitment process. Persons who are motivated to recruit patients to the study might best communicate information to potential participants. Future studies may benefit from putting more resources into the recruitment process. In order to identify potentially eligible patients, a screening procedure to assess ADL problems might overcome the challenges met in this study.

Follow-up data were collected from questionnaires sent out by mail. This could have turned out to be a limiting factor for the data collection, but only very few reminders had to be sent out. The primary reason for missing data in the follow-up period was related to death. Only 20 of the 118 participants withdrew from filling in the questionnaires for other reasons during the follow-up period. According to power calculations and the goal of including data of at least 144 patients in each group, we were very concerned about the potential risk of loss during follow-up, being it due to death or non-participation in the patient questionnaire. We wanted a long follow-up period, but decided not to make it longer than 8 weeks, considering a potentially large mortality – which turned out to be even larger than expected!

This research has important implications for future studies involving disabled cancer patients. To our knowledge only very few studies have examined the association between ADL and quality of life of cancer patients in a randomised controlled setting, and no studies have reported the feasibility of conducting an occupational therapy intervention programme based on meetings between cancer patients and occupational therapists in a randomised controlled trial.

Steinhauser et al. [36] pointed out the importance of distinguishing between conducting research in early and late stages of the cancer disease, since missing data among participants in the follow-up period are a well-known challenge. We find it important to conduct rigorous evaluations, even of paramedical interventions. The acceptance of the intervention in this study should encourage further studies within rehabilitation interventions for cancer patients.

## Conclusion

This study demonstrates that, although difficult and expensive, it is feasible to recruit cancer patients into an intervention programme addressing activities of daily living. A lower participation rate must be expected compared with drug intervention studies, and this fact should be incorporated in study designs. The procedures of recruitment, intervention and follow-up of this study turned out to be useful, from the initial identification of patients and their needs, to the implementation of the intervention and data collection during the follow-up period, but development of more comprehensive recruitment strategies is essential for future research.

## Competing interests

The authors report no competing interest. The authors alone are responsible for the content and writing of the paper.

## Authors’ contributions

LLJ, JS, DGH and KLC contributed to conception and study design. LLJ and JS obtained the funding. LLJ, JS, DGH and KLC wrote the protocol. LLJ, JS and DGH were responsible for recruitment of patients. LLJ collected and managed the data and performed the analysis. LLJ, JS, DGH and KLC contributed to the interpretation of data. LLJ drafted the first version of the manuscript. LLJ, JS, DGH and KLC critically reviewed, revised and supplemented the manuscript. All authors approved the final version. LLJ is the guarantor.

## Pre-publication history

The pre-publication history for this paper can be accessed here:

http://www.biomedcentral.com/1472-6963/14/194/prepub
